# Hallmarks of cancer in patients with heart failure: data from BIOSTAT-CHF

**DOI:** 10.1186/s40959-024-00246-w

**Published:** 2024-08-05

**Authors:** P. F. van den Berg, L. I. Yousif, G. Markousis-Mavrogenis, C. Shi, V. Bracun, J. Tromp, S. de Wit, Y. Appels, E. M. Screever, J. P. Aboumsallem, W. Ouwerkerk, D. J. van Veldhuisen, H. H. W. Silljé, A. A. Voors, R. A. de Boer, Wouter C. Meijers

**Affiliations:** 1 Department of Cardiology, University Medical Centre Groningen, Groningen, The Netherlands; 2 Department of Cardiology, Erasmus MC, Cardiovascular Institute, Thorax Center, Rotterdam, The Netherlands; 3 National Heart Centre Singapore, Singapore, Singapore; 4 Saw Swee Hock School of Public Health, National University of Singapore, Singapore, Singapore; 5 Department of Dermatology, Amsterdam UMC, University of Amsterdam, Amsterdam Infection & Immunity Institute, Amsterdam, The Netherlands; 6 Department of Cardiology, Thorax Center, Erasmus University Medical Center, P.O. Box 2040, Rotterdam, 3000CA The Netherlands

**Keywords:** Heart failure, Cancer, Cardio-oncology, Biomarkers

## Abstract

**Background:**

Within cardio-oncology, emerging epidemiologic studies have demonstrated a bi-directional relationship between heart failure (HF) and cancer. In the current study, we aimed to further explore this relationship and investigate the underlying pathophysiological pathways that connect these two disease entities.

**Methods:**

We conducted a post-hoc analysis in which we identified 24 Gene Ontology (GO) processes associated with the hallmarks of cancer based on 92 biomarkers in 1960 patients with HF. We performed Spearman’s correlations and Cox-regression analyses to evaluate associations with HF biomarkers, severity and all-cause mortality.

**Results:**

Out of a total of 24 GO processes, 9 biological processes were significantly associated with adverse clinical outcome. *Positive regulation of mononuclear cell proliferation* demonstrated the highest hazard for reaching the clinical endpoint, even after adjusting for confounders: all-cause mortality HR 2.00 (95% CI 1.17–3.42), *p* = 0.012. In contrast, *negative regulation of apoptotic process* was consistently associated with a lower hazard of reaching the clinical outcome, even after adjusting for confounders: all-cause mortality HR 0.74 (95% CI 0.59–0.95), *p* = 0.016. All processes significantly correlated with HF biomarkers, renal function and HF severity.

**Conclusions:**

In patients with HF, GO processes associated with hallmarks of cancer are associated with HF biomarkers, severity and all-cause mortality.

**Supplementary Information:**

The online version contains supplementary material available at 10.1186/s40959-024-00246-w.

## Introduction

Heart failure (HF) and cancer are the leading causes of death in the western world, with over 310,000 patients dying from HF and more than 600,000 patients dying from cancer annually, in the US alone [1, 2]. These two syndromes are more connected than initially thought: epidemiological studies have demonstrated that patients with HF are at an increased risk of developing incident cancer [3–6]. Moreover, the improvement in HF treatment and management was associated with a shift from HF-mortality to non-cardiovascular (CVD) mortality, such as cancer [7]. In addition, preclinical studies found that HF stimulates tumour growth in vivo [8–10]. These studies represent the multifactorial interplay between the two disease entities and the mechanisms mediating the reverse cardio-oncological link [11].

A number of shared risk factors and pathophysiological pathways, including obesity, smoking and (low-grade) inflammation explain the coexistence of cancer and HF [11–13]. Recently, the focus of this link has been expanded with HF-related pathophysiological mechanisms: research has shed light on the roles of angiotensin-II, β-adrenergic receptors and increased sympathetic activity in cancer development [11, 14]. In addition to the pathological processes in HF, the pathological processes in cancer have been more intensively studied, and have been summarized as the hallmarks of cancer [15–17]. These are considered key biological properties in oncology.

To gain a more profound understanding of the connection between HF and cancer, we examined biological processes (utilizing a panel of 92 biomarkers) associated with the firmly established hallmarks of cancer. [18] It is worth noting that these biomarkers were sourced from an Olink© panel, thus signifying that not all of them have been definitively recognized in the clinical milieu as explicit “tumour markers”, given that certain biomarkers trace their origins back to experimental contexts. We explored the correlations between these processes and HF biomarkers, renal function and HF severity. Furthermore, we evaluated with Cox-regression analyses the association with all-cause mortality.

## Materials and methods

### Study population

This is a post-hoc study of the “BIOlogy Study to TAilored Treatment in Chronic Heart Failure” (BIOSTAT-CHF) cohort. The BIOSTAT-CHF study included patients from 2010 to 2015 in 11 European medical centres. It was a prospective study, aimed to investigate how the (sub)optimal (up)titration of HF patients correlated with the composite outcome of (HF)-rehospitalization and death [19]. Patients aged ≥ 18 were included on a voluntary basis. Patients suffered from either new-onset or worsening HF, defined as a reduced (≤ 40%) left ventricular ejection fraction (LVEF) or increased plasma concentrations of cardiac biomarkers (BNP > 400 pg/mL or NT-proBNP > 2000 pg/mL) [19]. An extensive description of inclusion and exclusion criteria is presented elsewhere [19]. To prevent bias, we excluded all patients with prevalent cancer (*N* = 75) and all patients with missing biomarker associated with malignancy levels (*N* = 481) (see Supplementary Fig. 1 for a flowchart of the study population). The distinction between cardiovascular and non-cardiovascular mortality was adjudicated by the principal investigator of BIOSTAT-CHF and was based on the available medical records in the various registries of the participating centres; a full of list of event adjudication criteria is published elsewhere in literature. Ethical review was obtained from all respective institutional review boards of countries involved in BIOSTAT-CHF and all patients provided written informed consent [19]. 

### Data analyses

Plasma levels of 92 biomarker associated with malignancies (see Supplementary Table 1 for a full list of the biomarker associated with malignancies and their abbreviations) were measured by Olink^®^ Biosciences (Uppsala, Sweden), using a Proseek^®^ Oncology II multiplex^96 × 96^ proximity extension assay (PEA) panel. For analysis, calcium-ethylenediaminetetraacetic acid (EDTA)-plasma was used [20]. The Olink^®^ panel consists of a wide array of biomarker associated with malignancies expressed in various organs and disease processes, such as angiogenesis and immune response [21]. In PEA, antibodies are marked with oligonucleotides, and are pair-bound to their targets. These pairs subsequently bind to the target protein, and hybridize in pair-fashion when they are brought in close proximity [21, 22]. DNA polymerase is added, which leads to DNA polymerization, thus creating a distinct PCR target sequence [22]. Of note, PEA leads to arbitrary units, rather than absolute values.

### Biomarker associated with malignancies and biological gene ontology (GO) processes

The Gene Ontology (GO) database is a chief bioinformatics database, aimed to unify the (universal) genome and gene products [23, 24]. Within the GO database, three domains are identified: cellular component, molecular function, and biological process. The latter facilitates enrichment analyses, enabling identification of certain genes and or proteins that are overexpressed in a large set of data and may be associated with disease phenotypes [24]. Following methods described in previous studies, we imported the 92 biomarker associated with malignancies into gProfiler, an online toolset that enables enrichment analysis, to perform overrepresentation analysis [20, 25]. Subsequently, we selected the biological processes from GO that were linked and validated in literature to the hallmarks of cancer and used those processes for further analyses [18]. Three hallmarks of cancer (enabling replicative immortality, genome instability and mutation, deregulating cellular energetic) were not overrepresented in our data and were not available for further analyses (see Supplementary Table 2) [18]. In order to reduce the dimensionality of data, we performed principal component analysis, which enables reduction of data dimension by identifying the principal components that account for the greatest variability in the data. The resulting weighted score per process was used for Cox-regression analyses, in which we also adjusted according to the BIOSTAT-CHF mortality model: age, blood urea nitrogen, haemoglobin, NT-proBNP and beta-blocker use at baseline, and also added estimated glomerular filtration rate (eGFR) and Growth differentiation factor 15 (GDF-15) in a more extensive model [26]. 

### Statistical analysis

Normally distributed data are presented as mean ± standard deviation (SD), non-normally distributed data are presented as median with interquartile range (IQR). Categorical data are presented as N (%). Differences between groups were tested with either a Student’s T-test, analysis of variance (ANOVA) or χ^2^ test where appropriate. Cox-regression analyses were performed to examine the association of biological processes with the endpoint of all-cause mortality and were visualized with forest plots. Correlations between biological GO processes and HF biomarkers, renal function and HF severity were assessed with Spearman’s ρ. Data were processed and analysed with STATAse14 (StataCorp LP, College Station, Texas, United States of America) and R 4.1.3 (Foundation for Statistical Computing, Vienna, Austria) and a two-tailed *p*-value of ≤ 0.05 was considered statistically significant.

## Results

### Patient characteristics

Biomarkers associated with malignancy were available in 1960 patients, all of whom were included for present analyses. Patients had an average age of 69 ± 12 years, and 513 (26%) were female. The median body mass index (BMI) was 27.0 kg/m^2^ (IQR 24.2–30.8) and LVEF was 30% (IQR 25–36). Regarding cardiac biomarkers levels, NT-proBNP was 2606 (IQR 1155–5447), troponin T 30.7 (18.8–52.4) and GDF-15 was 2677.0 (1689.0-4431.0). eGFR was 60 mL/min/1.73m^2^ (IQR 44.5–78.2). A majority of the patients had a history of primary hypertension (1215 (62%)), followed by a history of smoking (958 (49%)), atrial fibrillation (887 (45%)) and myocardial infarction (732 (37%)). Close to one third of patients suffered from renal disease (540 (28%)). Almost all patients used loop diuretics (1952 (100%)), followed by beta-blockers (1632 (83%)) and ACEi/ARB (1421 (73%)) (Table 1).

**Table 1:** Baseline characteristics of patient population

Variable	BIOSTAT	
**Demographics**		
N	1960	
Age (years)	69 ± 12	
Sex (% women)	513 (26%)	
BMI (kg/m^2^)	27.0 (24.2–30.8)	
LVEF (%)	30 (25–36)	
**Laboratory**		
NT-proBNP (pg/mL)	2606.0 (1155.0-5447.0)	
Troponin T (pg/mL)	30.7 (18.8–52.4)	
GDF-15 (pg/mL)	2677.0 (1689.0-4431.0)	
eGFR (mL/min/1.73m^2^)	60.1 (44.5–78.2)	
**Medical history**		
Primary hypertension	1215 (62.0)	
Myocardial infarction	732 (37.3)	
Atrial fibrillation	887 (45.3)	
Stroke	185 (9.4)	
Renal disease	540 (27.6)	
Smoking		
Past	958 (49.0)	
Current	289 (14.8)	
**Medication at baseline**		
Loop diuretics	1952 (99.6)	
Beta-blocker	1632 (83.3)	
ACEi/ARB	1421 (72.5)	
MRA	1036 (52.9)	

*Abbreviations* BMI, body mass index; LVEF, left ventricular ejection fraction; NT-proBNP, N-terminal pro B-type natriuretic peptide; GDF-15, growth/differentiation factor 15; eGFR, estimated glomerular filtration rate; ACEi, angiotensin-converting enzyme inhibitor; ARB, angiotensin receptor blocker; MRA, mineralocorticoid receptor antagonist

Normally disturbed data are presented as mean ± SD, non-normally distributed data are presented as median (IQR) and categorical data are presented as N (%yes) unless otherwise specified

### Biological GO processes and clinical outcomes

#### All-cause mortality

**Fig. 1:**
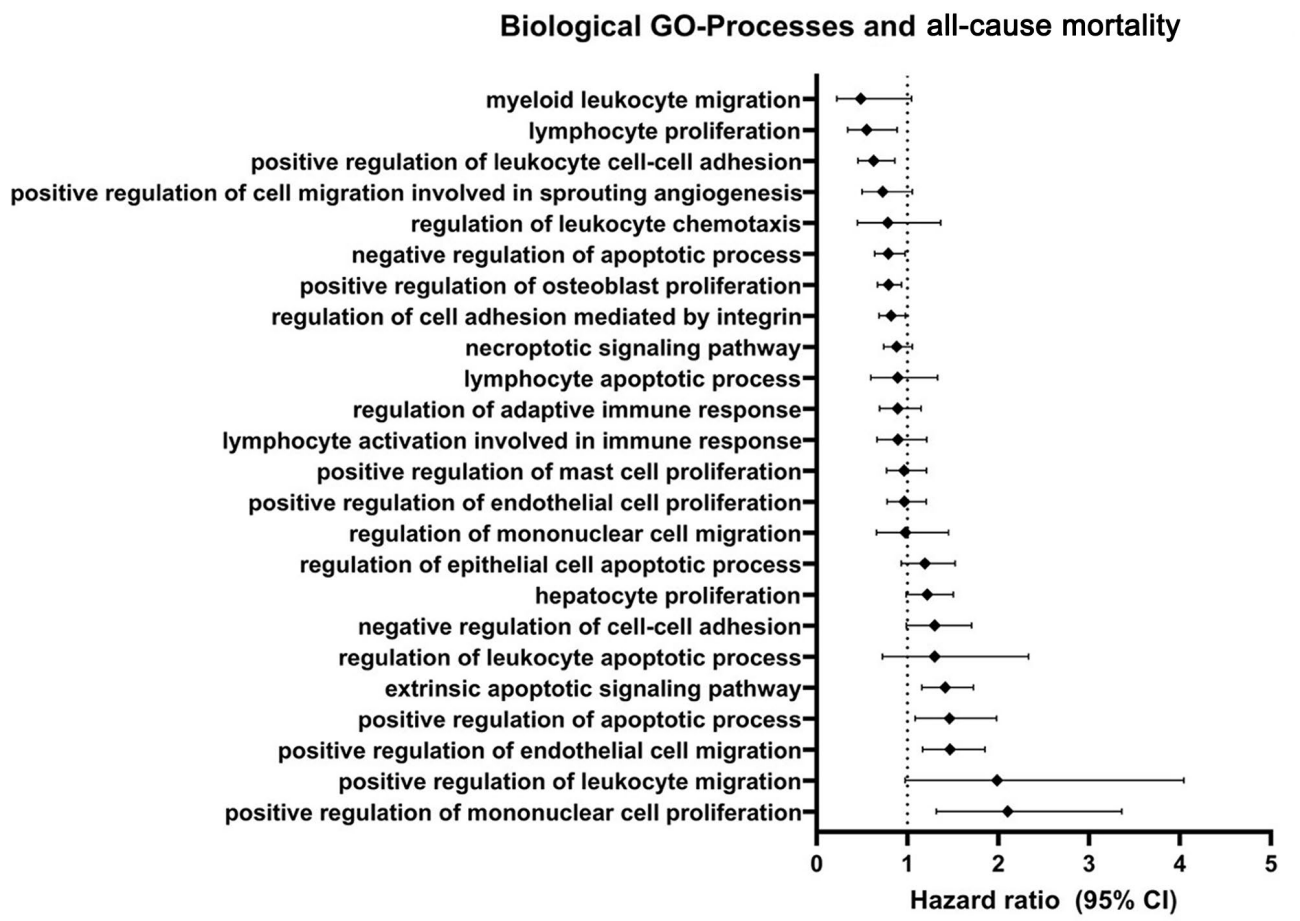
Forest plot demonstrating the association between biological GO-processes and association with all-cause mortality (ACM). A diamond indicates the hazard ratio (HR), and the line demarcated with vertical lines on either side represent the 95% confidence interval (CI).

Within two years, 451 patients reached the clinical outcome of all-cause mortality. In total, 24 biological processes were associated with the clinical outcome of all-cause mortality, 9 of which were significant (Fig. 1). Of these 9 processes, 4 exerted hazardous effects and 5 carried protective effects in unadjusted analysis (Table 2). After adjusting for confounders, 3 processes remained significantly associated with all-cause mortality. *Positive regulation of mononuclear cell proliferation* had the highest hazardous association (hazard ratio [HR] 2.00, 95% Confidence Interval (CI) 1.17–3.42, *p* = 0.012), followed by *extrinsic apoptotic signalling pathway* (HR 1.27, 95% CI 1.01–1.59, *p* = 0.038). *Negative regulation of apoptotic process* was associated with a lower hazard of reaching the clinical outcome of all-cause mortality (HR 0.74, 95% CI 0.59–0.95, *p* = 0.016).

**Table 2:** Cox-regression for biological GO-processes and associations with all-cause mortality

Biological Process	Model 1*		Model 2**		Model 3***	
	HR (95% CI)	*p*-value	HR (95% CI)	*p*-value	HR (95% CI)	*p*-value
**Positive regulation of mononuclear cell proliferation**	**2.11 (1.32–3.36)**	**0.002**	**1.98 (1.16–3.38)**	**0.012**	**2.00 (1.17–3.42)**	**0.012**
Positive regulation of leukocyte migration	1.99 (0.98–4.04)	0.058	1.97 (0.88–4.42)	0.102	1.92 (0.85–4.34)	0.116
**Positive regulation of endothelial cell migration**	**1.47 (1.17–1.85)**	**0.001**	**1.16 (0.89–1.50)**	**0.265**	**1.16 (0.90–1.51)**	**0.258**
**Positive regulation of apoptotic process**	**1.47 (1.08–1.98)**	**0.013**	**1.40 (0.99–1.97)**	**0.054**	**1.41 (1.00-1.99)**	**0.050**
**Extrinsic apoptotic signalling pathway**	**1.42 (1.16–1.73)**	**0.001**	**1.28 (1.03–1.60)**	**0.029**	**1.27 (1.01–1.59)**	**0.038**
Regulation of leukocyte apoptotic process	1.30 (0.72–2.33)	0.378	1.18 (0.60–2.31)	0.624	1.16 (0.59–2.29)	0.667
Negative regulation of cell-cell adhesion	1.30 (0.99–1.71)	0.058	1.07 (0.79–1.45)	0.641	1.06 (0.78–1.44)	0.692
Hepatocyte proliferation	1.22 (0.99–1.51)	0.066	1.24 (0.98–1.57)	0.078	1.22 (0.96–1.55)	0.112
Regulation of epithelial cell apoptotic process	1.19 (0.93–1.53)	0.158	0.96 (0.72–1.28)	0.782	0.95 (0.71–1.26)	0.708
Regulation of mononuclear cell migration	0.98 (0.66–1.45)	0.915	0.98 (0.62–1.54)	0.931	0.98 (0.62–1.55)	0.944
Positive regulation of endothelial cell proliferation	0.97 (0.77–1.21)	0.767	1.10 (0.85–1.41)	0.471	1.09 (0.85–1.41)	0.480
Positive regulation of mast cell proliferation	0.96 (0.77–1.21)	0.755	1.06 (0.81–1.38)	0.678	1.06 (0.81–1.39)	0.653
Lymphocyte activation involved in immune response	0.90 (0.66–1.21)	0.482	0.99 (0.71–1.37)	0.947	1.00 (0.72–1.40)	0.979
Regulation of adaptive immune response	0.89 (0.69–1.15)	0.380	0.96 (0.73–1.27)	0.782	0.96 (0.72–1.27)	0.751
Lymphocyte apoptotic process	0.89 (0.60–1.33)	0.578	0.94 (0.60–1.49)	0.806	0.95 (0.60–1.49)	0.814
Necroptotic signalling pathway	0.88 (0.74–1.05)	0.169	0.93 (0.76–1.14)	0.471	0.94 (0.76–1.14)	0.515
**Regulation of cell adhesion mediated by integrin**	**0.82 (0.69–0.98)**	**0.029**	**0.91 (0.74–1.11)**	**0.347**	**0.89 (0.73–1.01)**	**0.295**
**Positive regulation of osteoblast proliferation**	**0.79 (0.67–0.94)**	**0.006**	**0.95 (0.78–1.17)**	**0.642**	**0.95 (0.77–1.16)**	**0.592**
**Negative regulation of apoptotic process**	**0.79 (0.64–0.98)**	**0.029**	**0.73 (0.58–0.93)**	**0.009**	**0.74 (0.59–0.95)**	**0.016**
Regulation of leukocyte chemotaxis	0.78 (0.45–1.36)	0.388	0.81 (0.43–1.52)	0.513	0.82 (0.43–1.55)	0.538
Positive regulation of cell migration involved in sprouting angiogenesis	0.73 (0.50–1.05)	0.093	0.89 (0.58–1.37)	0.602	0.89 (0.57–1.37)	0.590
**Positive regulation of leukocyte cell-cell adhesion**	**0.63 (0.46–0.86)**	**0.004**	**0.74 (0.52–1.07)**	**0.107**	**0.75 (0.52–1.08)**	**0.124**
**Lymphocyte proliferation**	**0.55 (0.34–0.89)**	**0.014**	**0.59 (0.34–1.01)**	**0.052**	**0.59 (0.34–1.01)**	**0.054**
Myeloid leukocyte migration	0.49 (0.23–1.05)	0.065	0.53 (0.22–1.26)	0.148	0.54 (0.22–1.29)	0.163

*Abbreviations* HR, hazard ratio; CI, confidence interval

*Model 1: crude analysis

** Model 2: adjusted for BIOSTAT-CHF mortality model: age, NT-proBNP, haemoglobin, beta-blocker use at baseline, blood urea nitrogen

*** Model 3: adjusted for BIOSTAT-CHF mortality model: age, NT-proBNP, haemoglobin, beta-blocker use at baseline, blood urea nitrogen, eGFR and GDF-15

### Biological GO processes and clinical parameters of HF

**Fig. 2:**
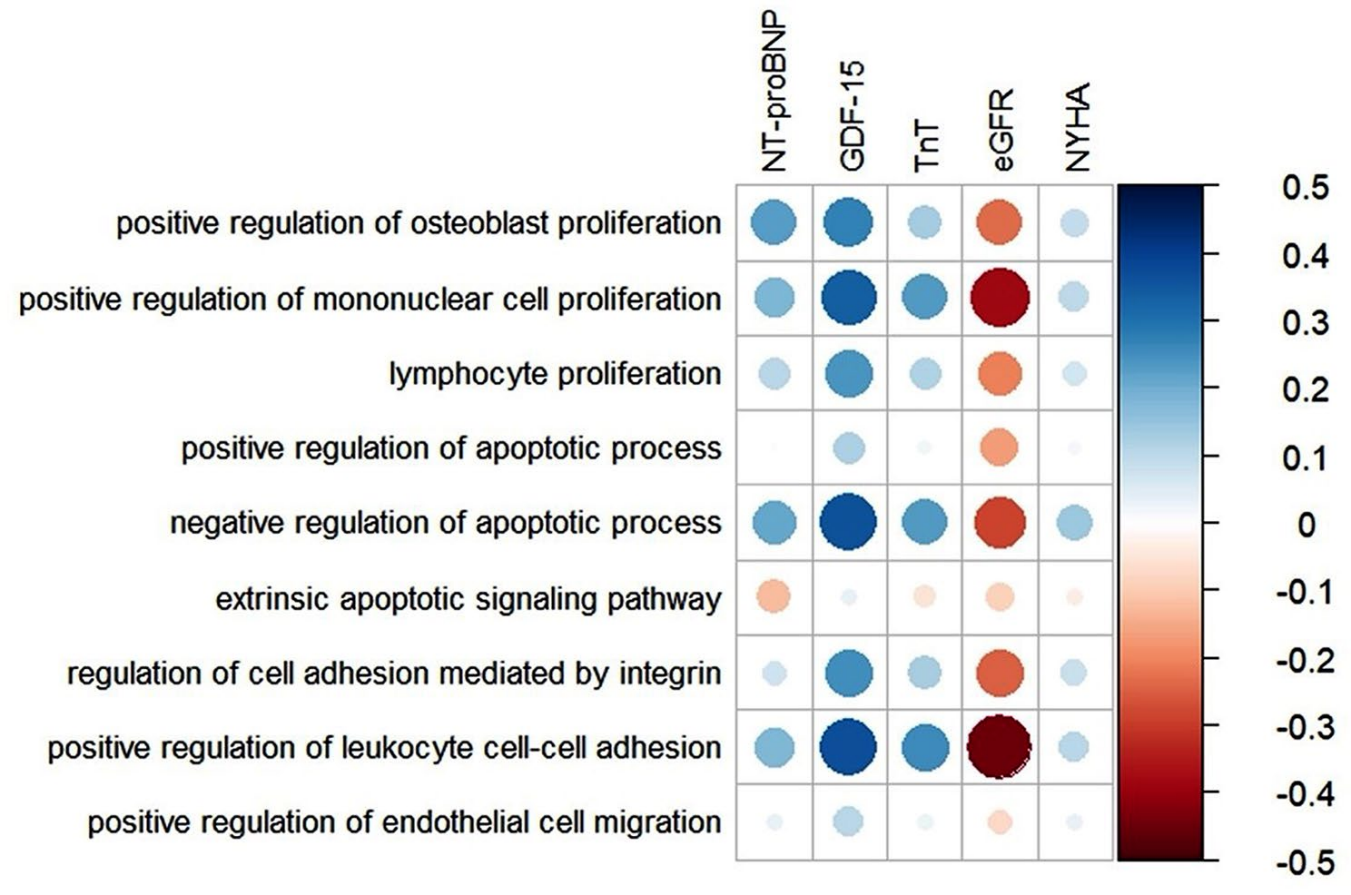
Correlation plot demonstrating the association between the nine biological GO-processes that were significantly associated with all-cause mortality and clinical parameters of HF. Positive and negative correlations are represented by blue and red, respectively, and the size of the circle indicates the strength of the correlation

Biological processes that were significantly associated with all-cause mortality, were also correlated with clinical parameters of HF (Fig. 2). *Positive regulation of mononuclear cell proliferation* and *negative regulation of apoptotic process* correlated positively with markers of HF (NT-proBNP, Troponin T, GDF-15) and NYHA-class, and were negatively correlated to eGFR. *Extrinsic apoptotic signalling pathway* was negatively correlated with NT-proBNP, Troponin T, eGFR and NYHA-class, but positively correlated with GDF-15.

## Discussion

### Main findings

In this study, based on a panel of 92 biomarker associated with malignancies, we investigated the association of hallmark biological processes in cancer with HF biomarkers, renal function, HF severity and all-cause mortality. We demonstrated both positive and negative associations between biological GO processes and all-cause mortality in patients with HF; *positive regulation of mononuclear cell proliferation* was associated with the highest hazard.

### GO processes and clinical outcome

Three GO processes showed an association with all-cause mortality, even after adjusting for the full BIOSTAT model. The highest hazard was associated with *positive regulation of mononuclear cell proliferation*. This process is in essence all that increases the regulation of mononuclear cell proliferation [27]. The role of blood peripheral mononuclear cells (PBMCs) (proliferation) has been studied extensively in the context of cancer [28]. In HF, the role of PBMC proliferation per se is less well known, but studies have postulated a link between CV disease and the interplay of PBMCs with the (innate) immune system and PBMC mitochondrial dysfunction [29, 30]. Interestingly, the lowest hazard was observed for *negative regulation of apoptotic process*. This process involves anything that reduces the extent of regulated cell death – apoptosis [31]. Apoptosis has been a key subject in cancer research for decades, and is seen as one of the promising targets for anticancer therapy [32]. Evading apoptosis is a hallmark of cancer, but in the setting of HF pathophysiology it has been more controversial [16, 33, 34]. Our study suggest that positive and negative regulation of apoptotic processes demonstrate both hazardous and protective associations, respectively. This further highlights the importance of apoptosis in HF. This is in line with recently published literature [35]. Lastly, it merits mentioning that several of the biomarkers and biological processes presented in this study are inflammation related, which have been extensively studied in this HF cohort. Therefore, we believe that to dive deeper into those processes would be beyond the scope of this study, in which we focus on the GO-processes that are well-established as hallmarks of cancer in a patient population with HF, but without overt cancer [20]. 

Nearly all processes demonstrated similar patterns regarding correlations with HF biomarkers, renal function and HF severity. The observation that processes associated with a higher hazard for the clinical outcome are also associated with higher levels of clinical markers of HF hints at the fact that HF may also lead to adverse events through other diseases (i.e., cancer pathogenesis) than solely HF.

### Future perspective

The challenge in the upcoming years will be to unravel the shared mechanisms between cancer and HF, and to uncover whether more severe HF (e.g., higher NT-proBNP levels, higher NYHA-classification) is associated with a more aggressive form of malignancy (e.g., higher tumour load, increased spread of metastases) or whether this relationship is unconditional, regardless of HF severity. In addition, it would be valuable to gain prospective or follow-up observations from present and future HF cohort studies to investigate the onset and pathogenesis of cancer in patients with HF, and the biological processes herein.

### Strengths and limitations

This study used an extensive and well-characterized cohort of patients with HF from multiple countries and added to the currently available data in the (translational) field of cardio-oncology, as no prior study has evaluated this large number of biomarkers associated with malignancies in an integrative approach. The biomarkers stemmed from a wide array of biological processes and tissues, thus covering a vast part of shared processes between HF and cancer. Lastly, all patients with cancer were excluded from analyses, ensuring that we only observe those processes associated with the hallmarks of cancer in patients with HF. However, besides the strengths of this study some limitations need to be addressed. First, the observational nature of this study renders it impossible to prove causality. Moreover, the databases that are used for pathway analyses rely on annotations from available scientific publications. This may cause overrepresentation of annotations that have been more intensively studied and more abundantly described in literature. Lastly, the endpoint of all-cause mortality rather than cause-specific mortality could have influenced the results as for example GDF-15 is a biomarker seen in a broad range of (inflammatory) disease (processes) and not solely bound to cancer and/or HF.

## Conclusion

In patients with HF, well-established biological processes linked to the hallmarks of cancer revealed 1) hazardous and protective associations regarding clinical outcome and 2) correlations with clinical parameters of HF. To improve our understanding of the complex interplay between HF and cancer, we call for further (prospective) translational research into the field of cardio-oncology that elaborate on the role of immunological processes in patients with HF at risk of developing cancer.

### Electronic supplementary material

Below is the link to the electronic supplementary material.

Supplementary Material 1

## Data Availability

The data that support the findings of this study are available from the BIOSTAT-CHF consortium, but restrictions apply to the availability of these data, which were used under license for the current study, and so are not publicly available. Data are however available from the authors upon reasonable request and with permission of BIOSTAT-CHF consortium.

## References

[1] Heidenreich PA, Bozkurt B, Aguilar D, Allen LA, Byun JJ, Colvin MM et al. 2022 AHA/ACC/HFSA Guideline for the management of Heart failure: a report of the American College of Cardiology/American Heart Association Joint Committee on Clinical Practice guidelines. Circulation. 2022;145(18). 10.1161/CIR.0000000000001063 35363499

[2] Siegel RL, Miller KD, Fuchs HE, Jemal A. Cancer statistics, 2022. CA Cancer J Clin. 2022;72(1):7–33. 35020204 10.3322/caac.21708

[3] Banke A, Schou M, Videbaek L, Møller JE, Torp-Pedersen C, Gustafsson F, et al. Incidence of cancer in patients with chronic heart failure: a long-term follow-up study. Eur J Heart Fail. 2016;18(3):260–6. 26751260 10.1002/ejhf.472

[4] Hasin T, Gerber Y, McNallan SM, Weston SA, Kushwaha SS, Nelson TJ, et al. Patients with heart failure have an increased risk of Incident Cancer. J Am Coll Cardiol. 2013;62(10):881–6. 23810869 10.1016/j.jacc.2013.04.088 PMC3758775

[5] Bertero E, Robusto F, Rulli E, D’Ettorre A, Bisceglia L, Staszewsky L, et al. Cancer Incidence and Mortality according to Pre-existing Heart failure in a community-based cohort. JACC CardioOncol. 2022;4(1):98–109. 35492831 10.1016/j.jaccao.2021.11.007 PMC9040106

[6] Aboumsallem JP, Moslehi J, de Boer RA. Reverse Cardio-Oncology: Cancer Development in patients with Cardiovascular Disease. J Am Heart Assoc. 2020;9(2). 10.1161/JAHA.119.013754 PMC7033852 31960736

[7] Boer RA, Meijers WC, Meer P, Veldhuisen DJ. Cancer and heart disease: associations and relations. Eur J Heart Fail. 2019;21(12). 10.1002/ejhf.1539 PMC6988442 31321851

[8] Meijers WC, Maglione M, Bakker SJL, Oberhuber R, Kieneker LM, de Jong S et al. Heart failure stimulates Tumor Growth by circulating factors. Circulation. 2018;138(7). 10.1161/CIRCULATIONAHA.117.030816 29459363

[9] Koelwyn GJ, Newman AAC, Afonso MS, van Solingen C, Corr EM, Brown EJ et al. Myocardial infarction accelerates breast cancer via innate immune reprogramming. Nat Med. 2020;26(9). 10.1038/s41591-020-0964-7 PMC7789095 32661390

[10] Avraham S, Abu-Sharki S, Shofti R, Haas T, Korin B, Kalfon R, et al. Early Cardiac Remodeling Promotes Tumor Growth Metastasis Circulation. 2020;142(7):670–83. 32475164 10.1161/CIRCULATIONAHA.120.046471

[11] Koelwyn GJ, Aboumsallem JP, Moore KJ, de Boer RA. Reverse cardio-oncology: exploring the effects of cardiovascular disease on cancer pathogenesis. J Mol Cell Cardiol. 2022;163:1–8. 34582824 10.1016/j.yjmcc.2021.09.008 PMC8816816

[12] Meijers WC, De Boer RA. Common risk factors for heart failure and cancer. Cardiovascular Res. 2019. 10.1093/cvr/cvz035 PMC6452432 30715247

[13] Boer RA, Hulot J, Tocchetti CG, Aboumsallem JP, Ameri P, Anker SD et al. Common mechanistic pathways in cancer and heart failure. A scientific roadmap on behalf of the Translational Research Committee of the Heart Failure Association (HFA) of the European Society of Cardiology (ESC). Eur J Heart Fail. 2020;22(12). 10.1002/ejhf.2029 PMC7894564 33094495

[14] Bertero E, Canepa M, Maack C, Ameri P. Linking heart failure to Cancer. Circulation. 2018;138(7):735–42. 30359132 10.1161/CIRCULATIONAHA.118.033603

[15] Hanahan D, Weinberg RA. The Hallmarks of Cancer. Cell. 2000;100(1):57–70. 10647931 10.1016/S0092-8674(00)81683-9

[16] Hanahan D, Weinberg RA. Hallmarks of Cancer: the Next Generation. Cell. 2011;144(5):646–74. 21376230 10.1016/j.cell.2011.02.013

[17] Hanahan D. Hallmarks of Cancer: New dimensions. Cancer Discov. 2022;12(1):31–46. 35022204 10.1158/2159-8290.CD-21-1059

[18] Chen Y, Verbeek FJ, Wolstencroft K. Establishing a consensus for the hallmarks of cancer based on gene ontology and pathway annotations. BMC Bioinformatics. 2021;22(1):178. 33823788 10.1186/s12859-021-04105-8 PMC8025515

[19] Voors AA, Anker SD, Cleland JG, Dickstein K, Filippatos G, van der Harst P et al. A systems BIOlogy Study to TAilored Treatment in Chronic Heart failure: rationale, design, and baseline characteristics of BIOSTAT-CHF. Eur J Heart Fail. 2016;18(6). 10.1002/ejhf.531 27126231

[20] Markousis-Mavrogenis G, Tromp J, Ouwerkerk W, Ferreira JP, Anker SD, Cleland JG et al. Multimarker profiling identifies protective and harmful immune processes in heart failure: findings from BIOSTAT-CHF. Cardiovasc Res. 2021. 10.1093/cvr/cvab235 PMC9239579 34264317

[21] Björkman K, Mustonen H, Kaprio T, Haglund C, Böckelman C. Mucin 16 and kallikrein 13 as potential prognostic factors in colon cancer: results of an oncological 92-multiplex immunoassay. Tumor Biology. 2019;41(7). 10.1177/1010428319860728 31264534

[22] Petrera A, von Toerne C, Behler J, Huth C, Thorand B, Hilgendorff A, et al. Multiplatform Approach for plasma proteomics: complementarity of Olink Proximity Extension Assay Technology to Mass Spectrometry-based protein profiling. J Proteome Res. 2021;20(1):751–62. 33253581 10.1021/acs.jproteome.0c00641

[23] Ashburner M, Ball CA, Blake JA, Botstein D, Butler H, Cherry JM, et al. Gene Ontology: tool for the unification of biology. Nat Genet. 2000;25(1):25–9. 10802651 10.1038/75556 PMC3037419

[24] The Gene Ontology Resource. 20 years and still GOing strong. Nucleic Acids Res. 2019;47(D1):D330–8. 30395331 10.1093/nar/gky1055 PMC6323945

[25] Raudvere U, Kolberg L, Kuzmin I, Arak T, Adler P, Peterson H, et al. G:profiler: a web server for functional enrichment analysis and conversions of gene lists (2019 update). Nucleic Acids Res. 2019;47(W1):W191–8. 31066453 10.1093/nar/gkz369 PMC6602461

[26] Voors AA, Ouwerkerk W, Zannad F, van Veldhuisen DJ, Samani NJ, Ponikowski P, et al. Development and validation of multivariable models to predict mortality and hospitalization in patients with heart failure. Eur J Heart Fail. 2017;19(5):627–34. 28247565 10.1002/ejhf.785

[27] GONUTS, Category. GO:0032946 ! positive regulation of mononuclear cell proliferation. 2020.

[28] Matthews HK, Bertoli C, de Bruin RAM. Cell cycle control in cancer. Nat Rev Mol Cell Biol. 2022;23(1):74–88. 34508254 10.1038/s41580-021-00404-3

[29] Sauer F, Riou M, Charles AL, Meyer A, Andres E, Geny B, et al. Pathophysiology of Heart failure: a role for peripheral blood mononuclear cells mitochondrial dysfunction? J Clin Med. 2022;11(3):741. 35160190 10.3390/jcm11030741 PMC8836880

[30] Alfatni A, Riou M, Charles AL, Meyer A, Barnig C, Andres E, et al. Peripheral blood mononuclear cells and platelets mitochondrial dysfunction, oxidative stress, and circulating mtDNA in Cardiovascular diseases. J Clin Med. 2020;9(2):311. 31979097 10.3390/jcm9020311 PMC7073649

[31] GONUTS, Category. GO:0043066 ! negative regulation of apoptotic process. 2020.

[32] Carneiro BA, El-Deiry WS. Targeting apoptosis in cancer therapy. Nat Rev Clin Oncol. 2020;17(7):395–417. 32203277 10.1038/s41571-020-0341-y PMC8211386

[33] Kang PM, Izumo S. Apoptosis and heart failure. Circ Res. 2000;86(11):1107–13. 10850960 10.1161/01.RES.86.11.1107

[34] Richter M, Kostin S. The failing human heart is characterized by decreased numbers of telocytes as result of apoptosis and altered extracellular matrix composition. J Cell Mol Med. 2015;19(11):2597–606. 26311501 10.1111/jcmm.12664 PMC4627565

[35] Aboumsallem JP, Shi C, De Wit S, Markousis-Mavrogenis G, Bracun V, Eijgenraam TR, et al. Multi-omics analyses identify molecular signatures with prognostic values in different heart failure aetiologies. J Mol Cell Cardiol. 2023;175:13–28. 36493852 10.1016/j.yjmcc.2022.12.001

