# Supplementary material for: Hallmarks of cancer in patients with heart failure: data from BIOSTAT-CHF
Source: Cardiooncology. 2024 Aug 5;10:47. doi: 10.1186/s40959-024-00246-w (PMC11299300; doi:10.1186/s40959-024-00246-w)

# **Supplementary Tables**

## **Supplementary Table 1. Overview of all 92 tumour-associated biomarkers.**

| **Abbreviation** | **Tumour-associated Biomarker** |
| --- | --- |
| ABL1 | Abelson murine leukemia viral oncogene homolog 1 |
| ADAM8 | A disintegrin and metalloproteinase domain-containing protein 8 |
| ADAMTS15 | A disintegrin and metalloproteinase with thrombospondin motifs 15 |
| ANXA1 | Annexin A1 |
| AREG | Amphiregulin |
| CAIX | Carbonic anhydrase 9 |
| CD160 | Cluster of differentiation 160 |
| CD207 | Cluster of differentiation 207 |
| CD27 | Cluster of differentiation 27 |
| CD48 | Cluster of differentiation 48 |
| CD70 | Cluster of differentiation 70 |
| CDKN1A | Cyclin-dependent kinase inhibitor 1 |
| CEA | Carcinoembryonic antigen |
| CEACAM1 | Carcinoembryonic antigen-related cell adhesion molecule 1 |
| CPE | Carboxypeptidase E |
| CRNN | Cornulin |
| CTSV | Cathepsin 5 |
| CXCL13 | C-X-C motif chemokine ligand 13 |
| CXCL17 | C-X-C motif chemokine ligand 17 |
| CYR61 | Cysteine-rich angiogenic inducer 61 |
| DLL1 | Delta-like 1 |
| EGF | Epidermal growth factor |
| EPHA2 | Ephrin type-A receptor 2 |
| ErbB2/HER2 | Receptor tyrosine-protein kinase erbB-2 |
| ErbB3/HER3 | Receptor tyrosine-protein kinase erbB-3 |
| ErbB4/HER4 | Receptor tyrosine-protein kinase erbB-4 |
| ESM1 | Endothelial cell-specific molecule 1 |
| FADD | Fas-associated protein with death domain |
| FasL | Fas ligand |
| FCRLB | Fc receptor-like B |
| FGFBP1 | Fibroblast growth factor-binding protein 1 |
| FRA | Folate receptor alpha |
| FRG | Folate receptor gamma |
| FUR | Furin |
| Gal1 | Galectin-1 |
| GPC1 | Glypican-1 |
| GPNMB | Transmembrane glycoprotein NMB |
| GZMB | Granzyme B |
| GZMH | Granzyme H |
| HGF | Hepatocyte growth factor |
| hK11 | Kallikrein 11 |
| hK14 | Kallikrein 13 |
| hK8 | Kallikrein 8 |
| ICOSLG | Inducible T-cell co-stimulator ligand |
| IFNGR1 | Interferon gamma receptor 1 |
| IGF1R | Insulin-like growth factor 1 |
| IL6 | Interleukin 6 |
| ITGAV | Integrin alpha-V |
| ITGB5 | Integrin beta-5 |
| KLK13 | Kallikrein-13 |
| LY9 | T-lymphocyte surface antigen Ly-9 |
| LYN | Tyrosine-protein kinase Lyn |
| LYPD3 | Ly6/PLAUR domain-containing protein 3 |
| MADH5 | Mothers against decapentaplegic homolog 5 |
| MetAP2 | Methionine aminopeptidase 2 |
| MIA | Melanoma-derived growth regulatory protein |
| MICAB | MHC class I polypeptide-related sequence A/B |
| MK | Midkine |
| MSLN | Mesothelin |
| MUC16 | Mucin 16 |
| NT5 | 5'-nucleotidase |
| PODXL | Podocalyxin-like protein 1 |
| PPY | Pancreatic prohormone Y |
| PVRL4 | Poliovirus receptor-related 4 |
| RET | RET proto-oncogene |
| RSPO3 | R-spondin-3 |
| S100A11 | S100 calcium-binding protein A11 |
| S100A4 | S100 calcium-binding protein A4 |
| SCAMP3 | Secretory carrier-associated membrane protein 3 |
| SCF | Stem cell factor |
| SEZ6L | Seizure 6-like protein |
| SPARC | Secreted protein acidic and rich in cysteine |
| SYND1 | Syndecan 1 |
| TCL1A | T-cell leukemia/lymphoma protein 1A |
| TFPI2 | Tissue factor pathway inhibitor 2 |
| TGFA | Transforming growth factor alpha |
| TGFR2 | Transforming growth factor beta receptor 2 |
| TLR3 | Toll-like receptor 3 |
| TNFRSF19 | Tumour necrosis factor receptor superfamily member 19 |
| TNFRSF4 | Tumour necrosis factor receptor superfamily member 4 |
| TNFRSF6B | Tumour necrosis factor receptor superfamily member 6B |
| TNFSF13 | Tumour necrosis factor superfamily member13 |
| TRAIL | Tumour necrosis factor-related apoptosis-inducing ligand |
| TXLNA | Alpha-taxilin |
| VEGFA | Vascular endothelial growth factor Alpha |
| VEGFR2 | Vascular endothelial growth factor receptor 2 |
| VEGFR3 | Vascular endothelial growth factor receptor 3 |
| VIM | Vimentin |
| WFDC2 | WAP four-disulfide core domain 2 |
| WIF1 | Wnt inhibitory factor 1 |
| WISP1 | WNT1-inducible-signaling pathway protein 1 |
| XPNPEP2 | Xaa-Pro aminopeptidase 2 |

## **Supplementary Table 2. Overview of biological GO-processes per hallmark of cancer (^*^ represents excluded hallmarks not represented in the data).**

| **Hallmarks of cancer** | **GO terms** | **Term name** |
| --- | --- | --- |
| Sustaining Proliferative Signaling | GO:0008283 | Cell Proliferation |
| Sustaining Proliferative Signaling | GO:0007049 | Cell Cycle |
| Sustaining Proliferative Signaling | GO:0051301 | Cell Division |
| Sustaining Proliferative Signaling | GO:0030307 | Positive regulation of cell growth |
| Sustaining Proliferative Signaling | GO:0045786 | Negative regulation of cell cycle |
| Sustaining Proliferative Signaling | GO:0008284 | Positive regulation of cell proliferation |
| Sustaining Proliferative Signaling | GO:0045787 | Positive regulation of cell cycle |
| Evading Growth Suppressor | GO:0009968 | Negative regulation of signal transduction |
| Evading Growth Suppressor | GO:0045786 | Negative regulation of cell cycle |
| Evading Growth Suppressor | GO:0008285 | Negative regulation of cell proliferation |
| Evading Growth Suppressor | GO:0030308 | Negative regulation of cell growth |
| Resist Cell Death | GO:0012501 | Programmed cell death |
| Resist Cell Death | GO:0043067 | Regulation of programmed cell death |
| Resist Cell Death | GO:0043069 | Negative regulation of programmed cell death |
| Enabling Replicative Immortality* | GO:0032200 | Telomere organization |
| Enabling Replicative Immortality* | GO:0001302 | Replicative cell aging |
| Enabling Replicative Immortality* | GO:2000772 | Regulation of cellular senescence |
| Enabling Replicative Immortality* | GO:0000723 | Telomere maintenance |
| Enabling Replicative Immortality* | GO:0032204 | Regulation of telomere maintenance |
| Enabling Replicative Immortality* | GO:0090398 | Cellular senescence |
| Inducing Angiogenesis | GO:0001525 | Angiogenesis |
| Inducing Angiogenesis | GO:0001570 | Vasculogenesis |
| Inducing Angiogenesis | GO:0045766 | Positive regulation of angiogenesis |
| Activating Invasion and Metastasis | GO:0034330 | Cell junction Organization |
| Activating Invasion and Metastasis | GO:0030030 | Cell projection Organization |
| Activating Invasion and Metastasis | GO:0030155 | Regulation of Cell Adhesion |
| Activating Invasion and Metastasis | GO:0007162 | Negative regulation of cell adhesion |
| Activating Invasion and Metastasis | GO:0007155 | Cell adhesion |
| Activating Invasion and Metastasis | GO:0016477 | Cell migration |
| Activating Invasion and Metastasis | GO:0001837 | Epithelial to mesenchymal transition |
| Genome Instability and Mutation* | GO:0045005 | Maintenance of fidelity involved in DNA-dependent DNA replication |
| Genome Instability and Mutation* | GO:0031570 | DNA integrity Checkpoint |
| Genome Instability and Mutation* | GO:0006281 | DNA repair |
| Genome Instability and Mutation* | GO:0006282 | Regulation of DNA repair |
| Tumour promoting Inflammation | GO:0002367 | Cytokine production involved in immune response |
| Tumour promoting Inflammation | GO:0050727 | Regulation of inflammatory response |
| Deregulating Cellular Energetic* | GO:0071456 | Cellular response to hypoxia |
| Deregulating Cellular Energetic* | GO:0006096 | Glycolysis |
| Avoiding Immune Destruction | GO:0002418 | Immune response to tumour cells |
| Avoiding Immune Destruction | GO:0006955 | Immune Response |
| Avoiding Immune Destruction | GO:0002837 | Regulation of immune response to tumour cell |
| Avoiding Immune Destruction | GO:0050776 | Regulation of immune response |

# **Supplementary Figures**

## **Supplementary Figure 1. Flowchart of study population.**


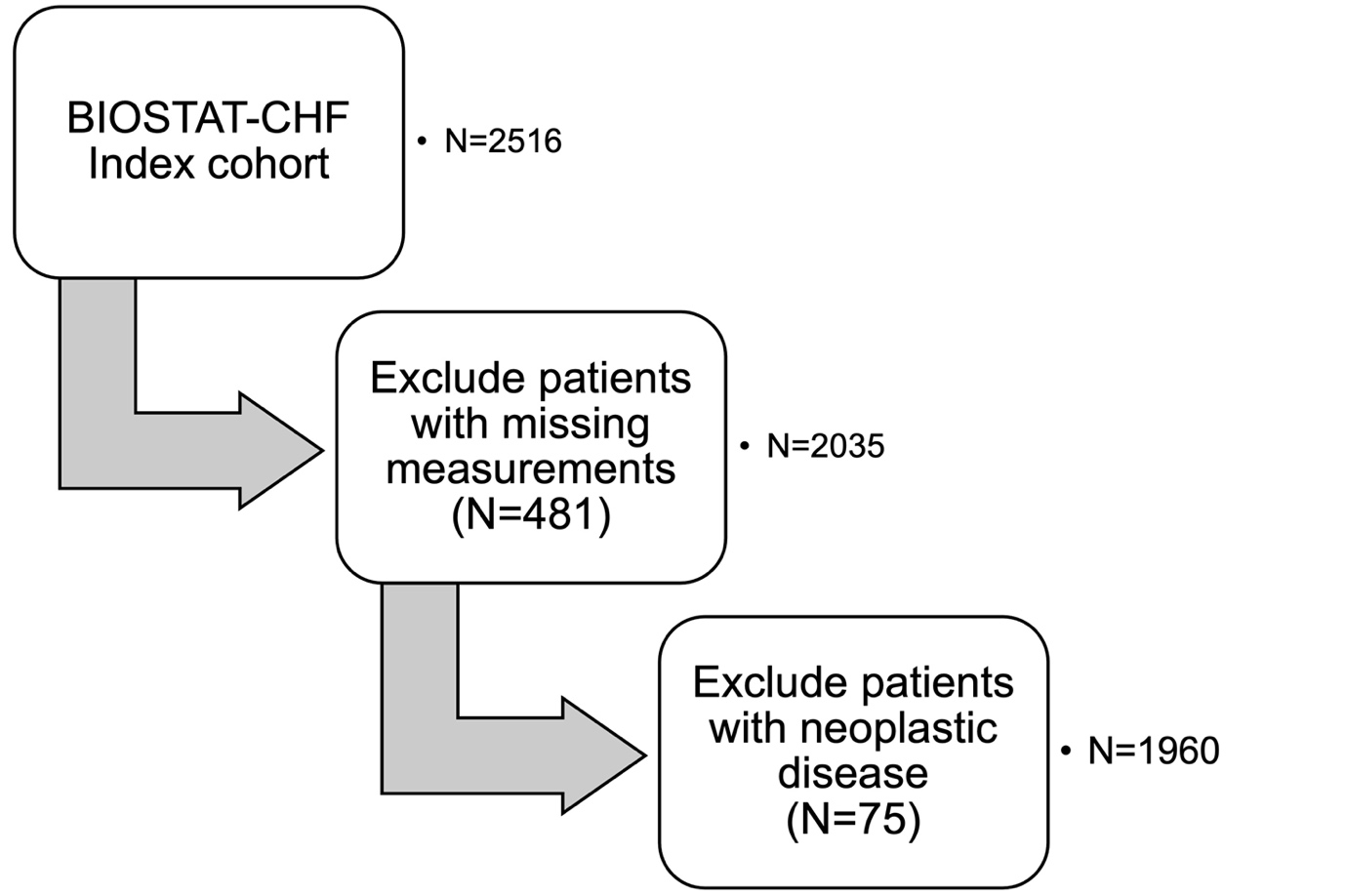

Supplement: Supplementary file 1 — Supplementary Material 1 [file 40959_2024_246_MOESM1_ESM.doc]
